# The efficacy and safety of *Shenzhu Guanxin* Recipe Granules for the treatment of patients with coronary artery disease: protocol for a double-blind, randomized controlled trial

**DOI:** 10.1186/s13063-019-3629-4

**Published:** 2019-08-20

**Authors:** Xiao Jin, Biqi Pan, Huanlin Wu, Bingxin Wu, Yukai Li, Xia Wang, Guoqing Liu, Xiaojing Dang, Danping Xu

**Affiliations:** 1 Second School of Clinical Medicine, Guangzhou University of Chinese Medicine, Guangzhou, China; 2 Department of Traditional Chinese medicine, GuangDong Women and Children Hospital, Guangzhou, China; 3 Dongzhimen Hospital Affiliated to Beijing University of Chinese Medicine, Beijing, China; 4 Department of Cardiology, Second Affiliated Hospital of Guangzhou University of Chinese Medicine (Guangdong Provincial Hospital of Chinese Medicine), Guangzhou, China

**Keywords:** Coronary artery disease, Traditional Chinese herbal, Randomized controlled trial, Intermediate coronary lesions

## Abstract

**Background:**

Coronary artery disease (CAD) is one of the most common types of the cardiovascular disease. Previous pilot trials have suggested that Traditional Chinese Medicine (TCM) has brought clinical benefits for patients with CAD. We will conduct this trial to determine the efficacy and safety of *Shenzhu Guanxin* Recipe Granules (SGR) for the treatment of patients with CAD.

**Methods:**

This randomized controlled trial recruited 190 patients who were diagnosed with CAD by clinical manifestation and examination and in which coronary computed tomography angiography (CCTA) showed 50–70% stenosis, with soft or mixed plaque types. The included participants were randomly assigned to the case group and control group using a 1:1 allocation ratio; patients in the case group received SGR and usual care, and those in the control group received placebo (6 g/day for 6 months) and usual care. The endpoint of the study included Calcium Coverage Score (CCS), C-reactive protein (CRP) level, and the levels of blood lipids, tumor necrosis factor-α (TNF-α), interleukin-1 (IL-1), interleukin-6 (IL-6), and ATP-binding membrane cassette transporter A1 (ABCA1) were calculated before recruiting and at the sixth month. The indicators were Seattle Angina Questionnaire (SAQ) and TCM Syndrome Questionnaire scores at 0, 3, and 6 months.

**Discussion:**

This clinical trial may provide reliable evidence regarding the clinical effectiveness and safety of SGR therapy for patients with CAD diagnosed by clinical manifestation and examination, in which CCTA showed 50–70% stenosis, with soft or mixed plaque types.

**Trial registration:**

ClinicalTrials.gov, ID: ChiCTR1900020501. The trial was registered on 25 December 2018.

**Electronic supplementary material:**

The online version of this article (10.1186/s13063-019-3629-4) contains supplementary material, which is available to authorized users.

## Introduction

Coronary artery disease (CAD) is one of the most common types of cardiovascular disease [1], resulting in over 9.5 million deaths worldwide [2], and has remarkably increased globally from 5.2 million deaths in 1990 [3]. Intermediate coronary lesions (ICL), which are defined as luminal narrowing with a stenosis diameter of 40–70%, continue to be a therapeutic dilemma for cardiologists [4]. Although previous studies have indicated that a stenosis diameter of 40–70% may be an indication for percutaneous coronary intervention (PCI), the treatment of ICL remains controversial [5, 6]. In non-left-main-stem lesions, the guidelines published by the American College of Cardiology (ACC)/American Heart Association (AHA) for PCI recommended ≥ 70% stenosis as the criterion for significant stenosis [7], while the guidelines released by the European Society of Cardiology expressed 50% stenosis with documented ischemia as the criterion for revascularization. Consequently, whether to recruit conservative treatment or adopt an aggressive revascularization strategy to perform PCI remains the main challenge for patients with ICL.

Regarding the possible side effects of PCI, some patients with ICL select a conservative treatment strategy. In addition, drugs play an important role in the conservative treatment of ICL. Although antiplatelet therapy and anticoagulation therapy are the cornerstones for treating coronary heart disease (CHD) [8], traditional Chinese herbal products possess several benefits for patients with CHD, especially the relief of clinical symptoms [9–11]. Based on the theory of Traditional Chinese Medicine (TCM), CHD belongs to the category of “chest pain” and “heart pain”, which is mainly caused by “*Qi* stagnation,” “blood stasis” and “phlegm turbidity.” *Shenzhu Guanxin* Recipe Granules (SGR), a traditional Chinese herbal product, including *Radix Ginseng*, *Rhizoma Atractylodis*, *Radix Notoginseng*, *Rhizoma Pinelliae*, *Hirudo medicinalis*, *Radix Panacis quinquefolium*, and *Folium Nelumbinis*, has been proved to accelerate blood circulation, enhance *qi*, and eliminate intravascular phlegm, all playing critical roles in the pathogenesis and progress of CAD in TCM theory [12, 13]. One previous study in southern China revealed that SGR was effective and safe to improve Seattle Angina Questionnaire (SAQ) and TCM symptoms (using the TCM Syndrome Questionnaire) scores, and decrease adverse events, such as death, restenosis and other emergency cases in patients receiving a standard Western medicine treatment after PCI [13].

Based on previous clinical and experimental evidence, we hypothesized that SGR may provide an alternative therapeutic strategy for patients with ICL who prefer to receive conservative treatment in lieu of an aggressive revascularization strategy. Therefore, we performed a multicenter, randomized, double-blind, parallel, placebo-controlled clinical trial to further investigate the efficacy of the aforementioned treatment strategy.

## Methods

**Fig. 1:**
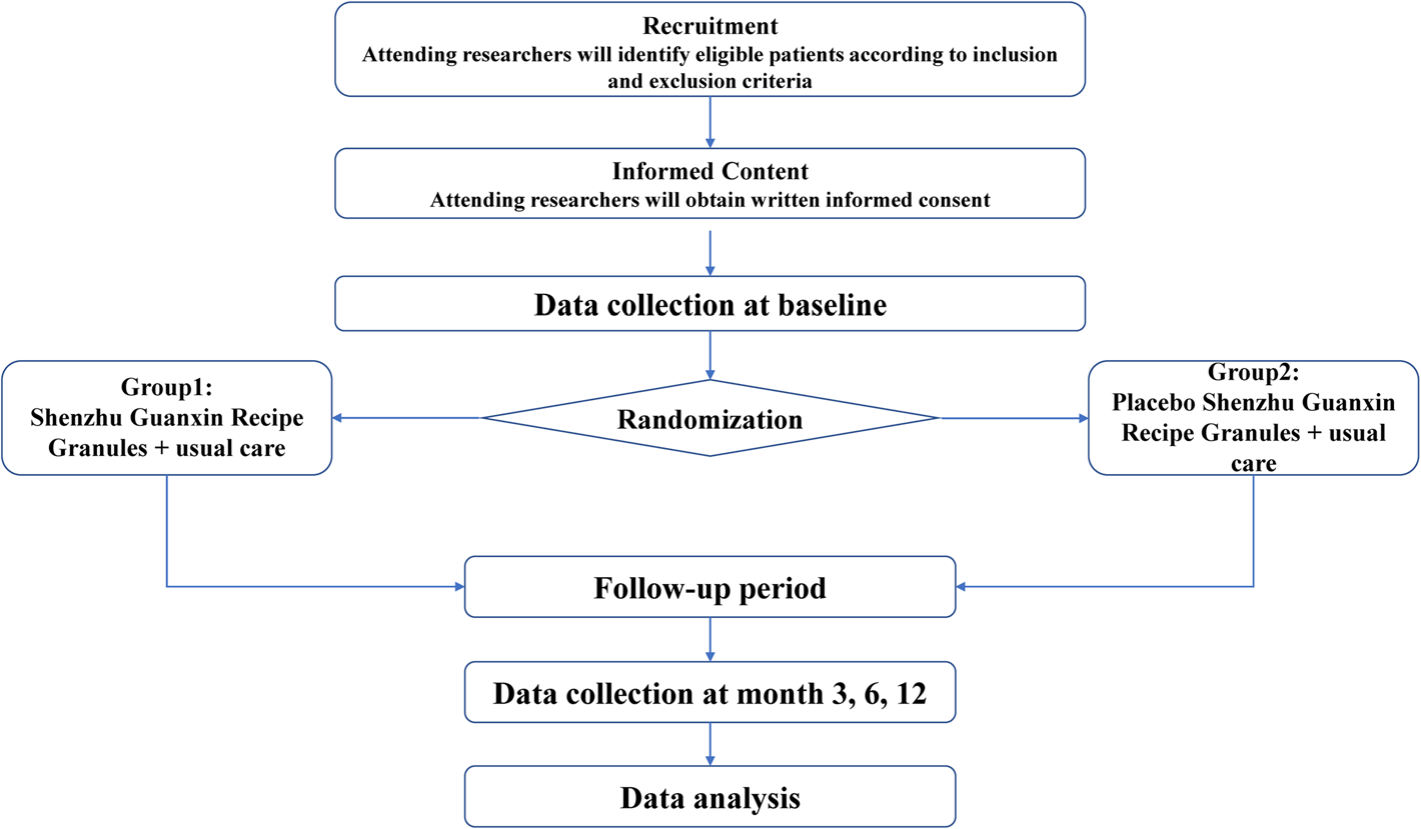
Flow diagram of the trial

This study mainly investigated the curative effects and possible mechanisms of SGR on CAD patients. In this study, patients with CAD were randomly assigned to a case group and a control group; the patients in the case group received SGR, and those in the control group received the placebo twice daily for 6 months. As soon as the patients were recruited to the trial, they underwent laboratory examinations and completed the SAQ. The laboratory examinations mainly included alanine amino transferase (ALT), aspartate amino transferase (AST), blood urea nitrogen (BUN), serum creatinine, and blood lipid levels. The patients were followed up for 12 months with repeated questionnaires every 3 months. At 6 months’ follow-up, the participants underwent repeated laboratory examinations, including coronary computed tomography angiography (CCTA). At the beginning of the study, all patients signed informed consent forms, in which they were informed about the risks and benefits of the treatment strategy. The study is guided by the Standard Protocol Items: Recommendations for Interventional Trials (SPIRIT) (Fig. 1 and Additional file 1). A flow diagram of the trial is shown in Fig. 1.

### Inclusion and exclusion criteria

The inclusion criteria were as follows: (1) the age of the subjects should be over 18 and under 75 years; (2) they should have been living in Guangdong province for a reasonable length of time (total duration of residency > 3 years, annual duration of residency > 9 months); (3) patients should be aware of the study and agree to sign an informed consent form; (4) patients should have been diagnosed with CAD by clinical manifestation and examination, and CCTA that showed 50–70% coronary atherosclerotic stenosis, with soft plaque or mixed plaque types; (5) the patients should not have undergo PCI surgery or coronary artery bypass grafting (CABG); and (6) phlegm and blood stasis syndrome (PBSS) due to the syndrome of *qi* deficiency should be evident. Syndrome differentiation will be determined by two qualified TCM cardiologists independently according to the diagnostic criteria for TCM differentiation (Table 1).

**Table 1:** Diagnostic criteria of Traditional Chinese Medicine (TCM) syndromes

Diagnostic criteria of TCM syndromes	
Main symptoms	1. Chest pain
	2. Sense of suppression in the chest
	The included patients need to have one of the main symptoms
*Qi* deficiency syndrome	1. Palpitation and shortness of breath
	2. Fatigue
	3. Spontaneous sweating
	4. Pale complexion
	5. Pale tongue
	6. Corpulent tender tongue with indentations in the margin of the tongue
	7. Weak pulse
	The included patients need to have two or more of the above symptoms
Blood stasis syndrome	1. Dark purple lips, complexions or claws
	2. Purple, dark or bruised tongue
	3. Enlarged sublingual venation
	4. Uneven pulse
	The included patients need to have two or more of the above symptoms
Phlegm turbidity syndrome	1. Being overweight
	2. Thick and greasy tongue
	3. Slippery pulse
	The included patients need to have two or more of the above symptoms

The exclusion criteria were as follows: (1) patients with complications of other serious diseases, including malignant tumors, severe infections, or other significant life-limiting comorbidities; (2) pregnant and lactating women and those allergic to TCM; (3) poorly compliant patients, and failure to visit regularly; (4) patients with valvular heart disease; (5) patients with congenital metabolic abnormalities or immune diseases; and (6) patients who have survival gains from revasularization, including those with two or three-vessel disease involving the proximal left anterior coronary descending branch (LAD) associated with the presence of either diabetes mellitus (DM) or a low ejection fraction (EF).

### Dropout criteria

The included patients have the right to stop treatment and withdraw from the research project for any reason at any time, and the reason that they want to quit the research project will be recorded in their case report form (CRF). Participants who do not complete the research project for the following reasons should be considered as having dropped out: (1) the patient chooses to quit the research project; (2) loss to follow-up; (3) poor compliance; (4) the participant develops another severe disease that needs to be treated during the study; and (5) patients whose symptoms, especially chest pain, are not relived during the study should exit and undergo a revascularization procedure.

### Recruitment strategies

In order to recruit patients, advertisements were placed in a broad range of media outlets, including flyers within the hospital, as well as the Chinese Clinical Trial Registry website. Patients who were interested in the trial received information about the study. Each potential participant was informed that the participation is fully voluntary and that refusal to participate in the research has no negative effect on their treatment. Those who would like to join the study were later assessed to determine whether they meet the inclusion criteria or not.

### Randomization and blinding

A random number generator in SAS 6.12 software (SAS Institute, Cary, NC, USA) was used by the DME (Design, Measurement, and Evaluation in Clinical Research) Center of Guangzhou University of Chinese Medicine (Guangzhou, China) to generate the random numbers in a 1:1 randomization ratio. The research team members, except for the clinical research methodology personnel, will be blinded to the treatment and the group assignment. Participants were informed about the information regarding the case group and the control group, while they were not told their group assignment, thereby allowing blinding of the participants between the treatment groups. Both the SGR and placebo granules were manufactured by the Jiangyin Tianjiang Pharmaceutical Co., Ltd. (Jiangyin, China), and the placebo was identical compared with the SGR in color, size, shape, and taste. The study code will not be revealed until the end of the study, unless a serious adverse event (AE) is reported.

### Intervention

After a recruitment period prior to baseline assessment, the included participants were randomized to the case group and control group, in which the patients in the case group received SGR and those in the control group received the placebo (6 g/day for 6 months). Simultaneously, the included patients also received usual care according to their conditions, including aspirin, clopidogrel, angiotensin-converting enzyme inhibitors or beta-blockers, calcium-channel blockers, and nitrate esters, irrespective of the initial randomization assignment. All the treatments were under the responsibility of physicians according to the clinical guidelines. Study medication (including both placebo and SGR) will be dispensed by the Hospital Central Pharmacy as a set of boxes at the beginning of each study month. In order to check the compliance of patients, all patients were asked to return the boxes. The treatment prescriptions and conditions of the patients were recorded in the CRF. Details of the study procedures of the trial are given in Table 2.

**Table 2:** Study procedures of the trial

Study procedures	Baseline	Treatment period		Follow-up period
Time point	0	3-month	6-month	12-month
Outcome measures				
Clinical data	√			
Coronary artery CT	√		√	
CRP	√		√	
Therapeutic evaluation				
Blood lipids	√		√	
SAQ	√	√	√	√
Scores of CM symptoms	√	√	√	√
ALT, AST	√		√	
Creatinine, blood urea nitrogen	√		√	
Safety indicators				
Blood glucose	√		√	
Drug interaction	√	√	√	√
Adverse event		√	√	√
Compliance observation		√	√	√
Medicine treatment	√	√	√	√

Note: *CRP* C-reactive protein, *CT* computed tomography, *SAQ* The Seattle Angina Questionnaire, *CM* Chinese medicine, *ALT* alanine aminotransferase, *AST* aspartate aminotransferase

### Outcome measures

Before starting the intervention, at 3 months after the intervention, after completing the intervention (6 months after treatment), and 1 year after the intervention, all the patients had to complete a questionnaire related to their quality of life. Additionally, all the patients underwent laboratory examinations and CCTA at baseline (prior to starting either intervention) and at 6 months’ follow-up. During the follow-up period, any enrolled participants who were unable to continue the study during the treatment remained in their randomized group to perform an intention-to-treat (ITT) analysis.

#### Primary outcomes

The primary endpoint of the study was CCTA, which mainly included the Calcium Coverage Score (CCS). The CCS represents the percentage of coronary arteries affected by calcific plaque, which was detected with cardiac computed tomography (CCT). The CCS has been shown to be reliable, reproducible, and predictive of cardiovascular risk, and also is highly associated with the risk of CAD [14–16]. One study revealed that a twofold increase in CCS was associated with a 34% (*P* < 0.001) increase in the risk of a hard CAD event, in addition to a 52% (*P* < 0.001) increase in the risk of any CAD event [17].

#### Secondary outcomes

Secondary outcomes were as follows: (1) concentration of ATP-binding membrane cassette transporter A1 (ABCA1), tumor necrosis factor-α (TNF-α), interleukin-1 (IL-1), and interleukin-6 (IL-6); (2) serum lipid levels; (3) the level of high-sensitivity C-reactive protein (CRP); (4) SAQ score; (5) TCM Syndrome Questionnaire score; and (6) evaluation of the occurrence and nature of major adverse cardiac events (MACE). The SAQ was used to assess the patients’ quality of life from five aspects, i.e., the level of limitation of physical activity, the severity of steady-state angina pectoris, condition of angina pectoris attacks, satisfaction level with treatments, and patient’s level of cognition regarding their disease [18]. The TCM Syndrome Questionnaire score included the following aspects: chest pain, sense of suppression in the chest, angina-attack inducement, and shortness of breath, fatigue, palpitation, and spontaneous perspiration [19] (Table 3).

**Table 3:** Primary and secondary outcomes

Primary endpoints	Coronary Artery Calcification Score, change of area of non-calcified plaque, and the proportion of non-calcified plaques to total plaques
Secondary endpoints	1. Concentration of gene *ABCA1*, and TNF-α, IL-1, IL-6
	2. Level of serum lipids
	3. Level of high-sensitivity C-reactive protein (CRP)
	4. Seattle Angina Questionnaire (SAQ) score
	5. Change of Traditional Chinese Medicine (TCM) syndrome
	6. Evaluation of occurrence and nature of major adverse cardiac events (MACE)

Note: *ABCA1* ATP-binding membrane cassette transporter A1, *TNF-α*  tumor necrosis factor-α, *IL-1* interleukin-1, *IL-6* interleukin-6

### Follow-up protocol

The first follow-up was carried out at 3 months after receiving treatment, and the patients’ health condition was assessed by inspecting the medical records, which were acquired by completing the CRF. The second follow-up was undertaken 6 months after receiving treatment, in which the eligible participants underwent CCTA and laboratory examinations, and the researchers also needed to assess the participants’ physical conditions according to the SAQ scores and scores of the TCM Syndrome Questionnaire. The third follow-up was performed at 12 months; the eligible participants had to complete the SAQ and the TCM Syndrome Questionnaire.

### Adverse events (AEs)

All drugs may have side effects or allergic reactions, although no adverse reactions from SGR have been reported yet. Any discomforts or unexpected situations that happen during the experiment period were regarded as AEs regardless of whether they were related to the study intervention or not. All the AEs were recorded in the CRF in detail. Serious AEs, including death, life-threatening or severely or permanently disabling events, were immediately reported to the principal investigator. The Ethics Committee assessed whether an AE was related to the experimental drug or not.

### Data management

To ensure strict adherence to the study protocol and familiarity with the trial administration process, an independent committee will be formed by the principal research members prior to the beginning of the study. Data management personnel of the committee should be qualified, effectively trained, and familiar with the functions of data management. A designated person is responsible for the data management of this clinical trial. When the patients are recruited to the research project, the demographic and baseline characteristic data will be collected by researchers. A standard CRF was used to collect data. Before the start of recording, data were de-identified. Clinical outcomes, the results of the SAQ and the TCM Syndrome Questionnaire, AEs, and the reasons why participants drop out of the study will be recorded in detail in the CRFs. In order to decease errors, CRF data will be entered by two researchers independently. They will check each other’s input values and only the consistent data can they be stored in the database. Paper files are kept in a locked filing cabinet in the hospital. With respect to the electronic documents, the results of laboratory tests and CCTA were stored on a password-protected computer, and access was restricted only to the principal investigator.

### Determination of the sample size

Firstly, we hypothesized that the expected difference in the primary outcome (Coronary Artery Calcification Score) between the SGR group and placebo group was estimated to be 10%. The reason that we considered 10% decrease as the clinically significant effect size was because a coverage probability of 90% for the confidence interval (CI) in the case of bioequivalence studies had become the accepted standard when evaluating whether the average values of the pharmacokinetic parameters of the two formulations were sufficiently close [20]. Thus, the 95% CI of the difference in the group means within the interval of − 10 to + 10% was defined as clinical equivalence in the current study. Secondly, to calculate the sample size, we employed the “pwr.t.test” function in R package “pwr” (R package version 1.2–2. https://CRAN.R-project.org/package=pwr) [21]. As an example, say we want to be able to detect a difference of at least 6.2 in the mean CCS (about 10% decreases in CCS) with a common standard deviation of the two groups to be 10. Therefore, our effect size is 6.2/10 = 0.62 according to Cohen (1988) [22]. For a desired power of 80%, Type I error tolerance of 0.05, and a hypothesized effect size of 0.62, we should sample at least 84 participants per group, i.e., a total of 168 participants. If we assume that there will be a dropout rate of 15% within 6 months, then 194 participants can eventually be recruited.

### Statistical analysis

All data analysis will be conducted by qualified statisticians in a double-blind manner according to the ITT principle. The database will be built by EpiData 3.1 software. In this study, SPSS 22.0 software (IBM, Armonk, NY, USA) was used to perform statistical analysis. Continuous variables were expressed as mean ± standard deviation (SD) or median, and categorical variables were reported as numbers and percentages. Student’s *t* test was also used for making comparison between the two groups. Additionally, one-way analysis of variance (ANOVA) was applied for making comparison between the groups. Pearson’s chi-squared test was applied to sets of categorical data to evaluate how likely it is that any observed difference between the sets arose by chance. A *P* value < 0.05 was considered statistically significant.

## Discussion

Although the treatment of CAD has advanced remarkably, the treatment of ICL remains controversial. Chinese herbs had been proved to have definite curative effects on CAD and have gradually attracted scholars’ attention in clinical trials [23–25]. Furthermore, SGR has been proved to be effective for patients with angina pectoris after PCI, and it can also upregulate the expression of platelet/endothelial cell adhesion molecule-1 (PECAM-1)/CD31 and vascular endothelial growth factor (VEGF), thereby promoting myocardium angiogenesis in myocardial infarction induced in rats [26]. In this study, we attempted to further investigate the efficacy and safety of SGR in patients with ICL, in which CCS was used as an indicator to evaluate its efficacy. The blood profile of our subjects was associated with atherosclerosis, which was one of the most important risk factors of CAD. The serum concentration of high-density lipoprotein-cholesterol (HDL-C) was detected as a strong, independent, and inverse predictor of atherosclerotic cardiovascular disease (ASCVD), in particular, CAD [27, 28]. Genes had been proved to be associated with the serum concentration of HDL-C, including *ABCA1*, which can modulate the concentration of HDL-C and catalyze the transfer of lipids from various tissues and cells to apolipoprotein A-I (apo A-I) [29, 30]. In this trial, in order to further reveal whether SGR can affect the metabolism of blood lipids, the concentration of *ABCA1* was also calculated in addition to the concentration of blood lipids.

Numerous studies have reported a relationship between the elevated levels of circulating inflammatory markers and adverse cardiovascular events [31]. TNF-α induces the production of IL-6, that can activate the hepatocyte production of CRP [32]. Among myriad inflammatory markers, CRP was found to be a valuable biomarker in refining risk assessment [33, 34]. In the present clinical trial, the influence of SGR on inflammatory markers, such as TNF-α, IL-1, IL-6, and CRP was also assessed.

This study not only evaluated the efficacy and safety of SGR therapy for patients with ICL, but also discussed the potential mechanisms of SGR for the treatment of CAD. To minimize bias of the trial, a rigorous set of methods was conducted, including randomization, in which the blinding method and statistical analysis were carried out according to ITT.

There are some limitations in this study. Firstly, although we recruited patients with PBSS, however, this Chinese medicine syndrome can dynamically change after the intervention, the patients will take SGR for 6 months even when the TCM syndrome may changed, and we did not take this point in this study. Secondly, the calculation of sample size in our study was based on a pilot study and clinical observations, maybe a larger sample size and multi-center study may result in different achievements. Third, even though several endpoints had been included in our trial to evaluate the efficacy of SGR on patients with CAD, and most of the patients in our study had already undergone cardiac echocardiography, the indicators of color Doppler echocardiography were not listed as endpoint indicators in our study. In order make the trial more rigorous and comprehensive, we should consider gathering and providing other important baseline data, such as ejection fraction (EF), which may affect the treatment protocols significantly in our future studies.

In summary, the present study has provided a solid foundation for the clinical treatment of CAD, in which further evidence can be achieved regarding the application of TCM for the treatment of CAD.

## Trial status

This research has been registered at the Chinese Clinical Trial Registry (No. ChiCTR1900020501), and the first patient in this trial was enrolled on 1 January 2015. The trial has already enrolled some participants: 50 patients have been recruited, and 40 patients have completed. The recruitment will be completed by 12 December 2019.

## Additional file

Additional file 1 Standard Protocol Items: Recommendations for Interventional Trials (SPIRIT) 2013 Checklist. (DOC 139 kb)

## Data Availability

Data sharing is not applicable to this article because no datasets were generated or analyzed during the study.

## Abbreviations

ABCA1: ATP-binding membrane cassette transporter A1
ANOVA: Analysis of variance
CABG: Coronary artery bypass grafting
CAD: Coronary artery disease
CCS: Calcium Coverage Score
CCTA: Coronary computed tomography angiography
CRP: C-reactive protein
HDL-C: High-density lipoprotein-cholesterol
ITT: Intention-to-treat
MACE: Major adverse cardiac events
PCI: Percutaneous coronary intervention
SAQ: Seattle Angina Questionnaire
SGR: *Shenzhu Guanxin* Recipe Granules
TCM: Traditional Chinese Medicine
TNF-α: Tumor necrosis factor-α
